# TUNEL Assay and DAPI Staining Revealed Few Alterations of Cellular Morphology in Naturally and Artificially Aged Seeds of Cultivated Flax

**DOI:** 10.3390/plants7020034

**Published:** 2018-04-13

**Authors:** Yong-Bi Fu, Zaheer Ahmed, Hui Yang, Carolee Horbach

**Affiliations:** Plant Gene Resources of Canada, Saskatoon Research and Development Centre, Agriculture and Agri-Food Canada, 107 Science Place, Saskatoon, SK S7N 0X2, Canada; zahmed758@gmail.com (Z.A.); huiyang1228@gmail.com (H.Y.); Carolee.horbach@agr.gc.ca (C.H.)

**Keywords:** flax, seed viability, seed aging, TUNEL, DAPI, seed cellular morphology

## Abstract

In a search for useful seed aging signals as biomarkers for seed viability prediction, we conducted an experiment using terminal deoxynucleotidyl transferase mediated dUTP nick end labeling (TUNEL) assay and 4′,6-diamidino-2-phenylindole (DAPI) staining to analyze morphological and molecular changes in naturally aged (NA) and artificially aged (AA) flax (*Linum usitatissimum* L.) seeds. A total of 2546 sections were performed from 112 seeds of 12 NA and AA seed samples with variable germination rates. Analyzing 1384 micrographs generated from TUNEL assay and DAPI staining revealed few alterations of the cellular morphology of the NA and AA seeds. Also, the revealed DNA degradations in the aged flax seeds appeared to be associated with seed samples of low germination rates. These results suggest that oily flax seed aging may alter the cellular morphology differently than starchy wheat seed aging. The results also imply that the TUNEL assay and DAPI staining may not yield informative assessments on cellular alterations and DNA degradation after the aging of oily seeds.

## 1. Introduction

Monitoring seed viability during long-term storage is an important and challenging task for the management and conservation of ex situ stored seeds in gene banks, as the unpredictable loss of seed viability during storage occurs and can lead to the depletion of valuable plant genetic resources [1,2]. Currently, a germination test is the standard method used to assess the viability of ex situ conserved seeds [1,3]. However, it can be a time-consuming and labor-intensive operation and does not provide an assessment of the underlying mechanisms of seed deterioration or any early projection of seed longevity for regeneration timing. Therefore, more informative tools are needed to supplement the standard germination test for more effective monitoring of seed aging in storage [1].

Seed aging or seed deterioration is commonly described as the loss of seed quality or viability over time [4,5]. It is a complex biological trait and involves a network of molecular, biochemical, physiological, and metabolic processes. Large efforts have been made to understand these aging processes from the aspects of seed development, vigor, viability, longevity, and germination (e.g., see [6,7,8,9]). Many reviews have been published attempting to explain various aspects around the progression of seed deterioration (e.g., see [10,11,12]). Generally, seed viability loss is associated with changes in cellular membrane damage, weakened energy metabolism, production of reactive oxygen species (ROS) and their counter balance, lipid peroxidation, compromised RNA and protein synthesis, inactivation of enzymes, and DNA degradation [4,7,8,9,13,14,15]. Some of these changes, such as DNA degradation, were the consequences of active and genetically controlled programmed cell death (PCD) [16]. However, the underlying biological mechanisms that regulate and execute PCD during seed aging remain largely unknown [17,18,19].

One of the widely used approaches for detecting DNA degradation and visualizing cellular alteration is terminal deoxynucleotidyl transferase mediated dUTP nick end labeling (TUNEL) assay coupled with 4′,6-diamidino-2-phenylindole (DAPI) staining [20]. DAPI staining can reveal the nuclei of living or dead cells by emitting blue fluorescence upon binding to AT regions of DNA [21]. TUNEL staining can detect DNA degradation through the enzyme terminal deoxynucleotidyl transferase (TdT) to incorporate labeled dUTP into free 3′-hydroxyl termini created during the fragmentation of genomic DNA, a salient feature of PCD [22]. TUNEL assay can also be applied to detect DNA impairment associated with non-apoptotic events, such as necrotic cell death induced by exposure to toxic compounds and radiations [23], and to stain cells undergoing active DNA repair [24]. Successful applications of TUNEL assay have been reported to study PCD and cellular alterations in the aged seeds of sunflowers (*Helianthus annuus* L.) [25], elm (*Ulmus pumila* L.) [26], and peas (*Pisum sativum* L.) [27].

To search for useful seed aging signals as biomarkers for seed viability prediction, we reviewed seed aging signals of different natures and explored their potential as tools for monitoring seed deterioration [1]. To continue the exploration empirically, we have also conducted a series of experiments with the aim to search for specific seed aging signals for seed viability prediction. So far, we have found the associative changes in scutellum nuclear content and morphology with viability loss in naturally aged (NA) and artificially aged (AA) wheat (*Triticum aestivum* L.) seeds [28]. In a separate simple sequence repeat (SSR) study, however, the assayed SSR variability did not show any association with seed viability loss in bread wheat seeds under NA and AA treatments [29]. This study represents another companion experiment with the aim to assess morphological and molecular changes in NA and AA flax (*Linum usitatissimum* L.) seeds using TUNEL assay and DAPI staining and to determine the association of the changes, if any, with seed germination. It was reasoned that the changes in the nuclear content and cellular morphology of aged flax oily seeds may differ from those in aged wheat seeds containing high levels of starch. 

## 2. Results

### 2.1. Seed Viability under NA and AA Treatments

Seed samples N95 and N95a exhibiting 95% viability were used as negative controls for comparison. Samples N90 and N91 were stored at −20 °C since 2000 and 1998 and exhibited 90% and 91% viability, respectively (Table 1). Samples N32, N22, and N33 were stored at 4 °C since 1998 and displayed viability of 32%, 22%, and 33%, respectively. Sample N3 was stored at 4 °C since 1973 and had only 3% viability. For the two control samples, N95 and N95a, considerable reduction in viability was observed following AA treatments (Table 1). These samples, when exposed to the AA treatment for 96 h at 43 °C, displayed viability reduction from 95% to 54% and 44%, respectively. When these samples were exposed to more drastic AA treatment (120 h at 44 °C), a severe reduction in viability (47% and 12%, respectively) was observed. These results show that both NA and AA treatments caused reduction in flax seed viability. The AA treatments were more drastic than the NA treatments, which were gradual and progressive. The impact of AA treatment was also sample-dependent; sample N95a had a lower germination rate (12%) than sample N95 (47%) when both were exposed to the same AA treatment (120 h at 44 °C) (Table 1). 

### 2.2. Assessment of Cellular Alterations Following NA and AA Treatments

**Figure 1:**
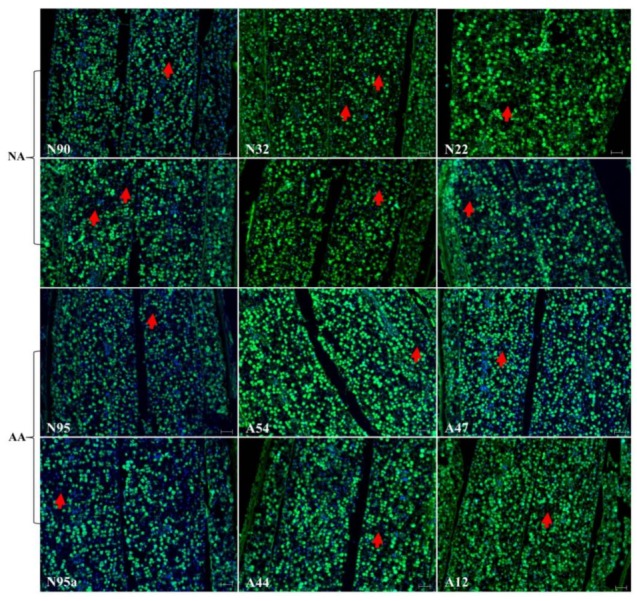
Illustration of no marked alterations in the cellular morphology in the embryonic sections of the aged flax seeds labeled with TUNEL assay and corresponding DAPI staining, although the disappearance of some nuclei (marked with red arrows) was observed. The top six panels represent the seeds under natural aging, while the bottom six panels represent the seeds under artificial aging. Each panel has a sample label with its aging treatment (N for natural aging or A for artificial aging) and germination rate (see Table 1), along with the scale bar of 50 µm. Note that the N95 and N95a panels are control samples before aging. TUNEL assay should show a green dot if degraded DNA within a nucleus is detected, while DAPI staining will display a blue dot if a nucleus is detected.

The experiment generated 2546 sections from 112 aged seeds, and the number of sections per seed sample ranged from 126 to 240 (Table 1). After TUNEL assay and DAPI staining, 1384 micrographs were produced, and the number of micrographs per seed sample ranged from 54 to 194 (Table 1). Detailed examinations of these micrographs for embryonic sections revealed no marked changes in the cellular morphology in the embryonic sections of the assayed seeds, regardless of their aging treatments, as illustrated in Figure 1. Blue dots generated by DAPI staining for nuclei of living or dead cells can be easily identified in each micrograph, but it was difficult to detect green dots generated by TUNEL assay for degraded DNA within the nuclei, probably due to the presence of numerous oil and protein bodies in the cells. The disappearance of some nuclei was observed, but such cell death did not seem to alter the overall cellular morphology of the aged seeds.

**Figure 2:**
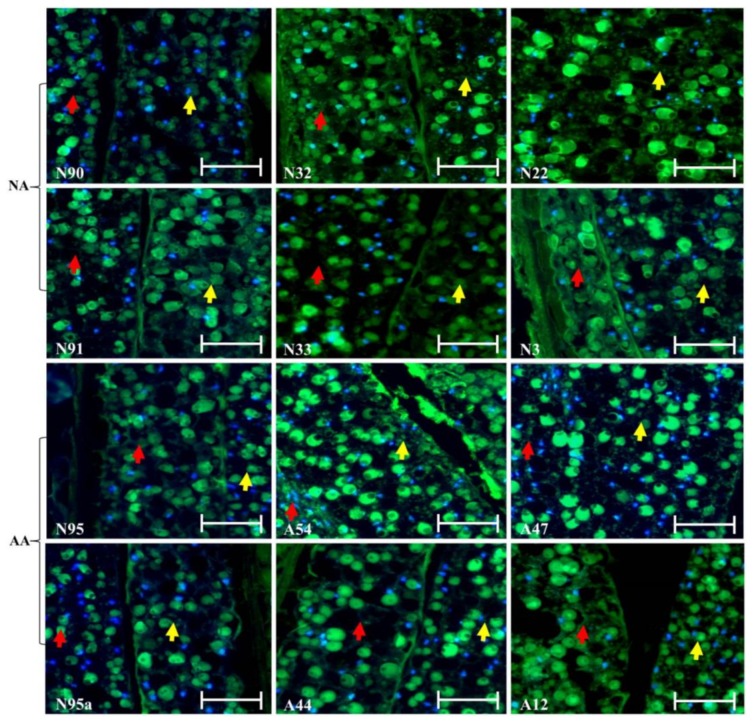
Illustration of no marked alterations of the cellular morphology in the endosperm and cotyledon sections of the aged flax seeds labeled with TUNEL assay and corresponding DAPI staining. The top six panels represent the seeds under natural aging, while the bottom six panels represent the seeds under artificial aging. Each panel has a sample label with its aging treatment (N for natural aging or A for artificial aging) and germination rate (see Table 1), along with the scale bar of 50 µm. Note that the N95 and N95a panels are control samples before aging. The red arrow is for the endosperm section, while the yellow arrow is for the cotyledon section.

**Figure 3:**
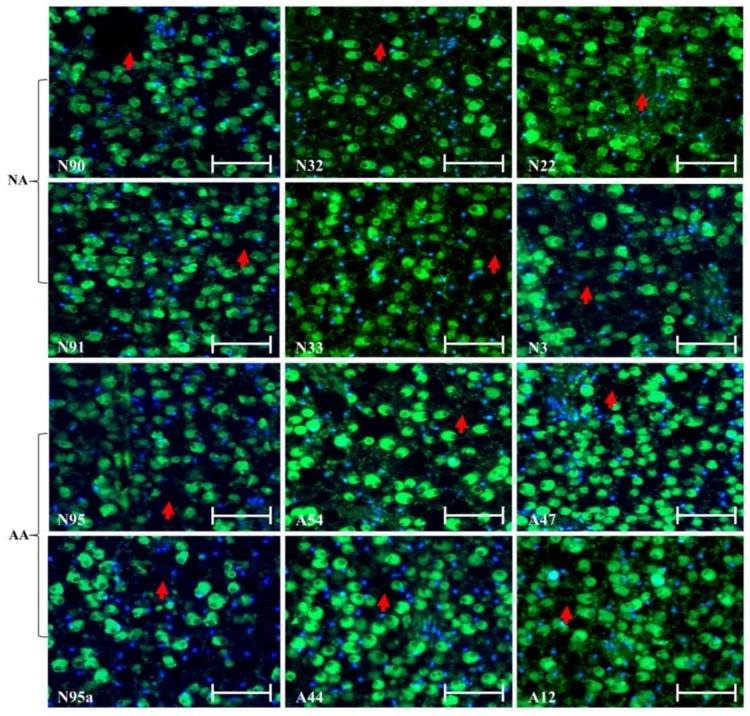
Illustration of no marked alterations of the cellular morphology in the radicle sections of the aged flax seeds labeled with TUNEL assay and corresponding DAPI staining. The top six panels represent the seeds under natural aging, while the bottom six panels represent the seeds under artificial aging. Each panel has a sample label with its aging treatment (N for natural aging or A for artificial aging) and germination rate (see Table 1), along with the scale bar of 50 µm. Note that the N95 and N95a panels are control samples before aging. The red arrows show nucleus disappearances.

Continuous assessment of the micrographs for endosperm and cotyledon sections revealed few changes in the cellular morphology in these sections of the assayed seeds (Figure 2). The same difficulty existed in identifying green dots for degraded DNA within the nuclei. Further micrograph evaluation of the radicle section of the NA and AA seeds revealed no marked alterations of the cellular morphology in this section of the assayed seeds, as illustrated in Figure 3. Some nuclei were also found to have disappeared, but degraded DNAs within the nuclei were hardly identified. Clearly, few alterations of the cellular morphology were found in these assayed NA and AA seeds.

**Figure 4:**
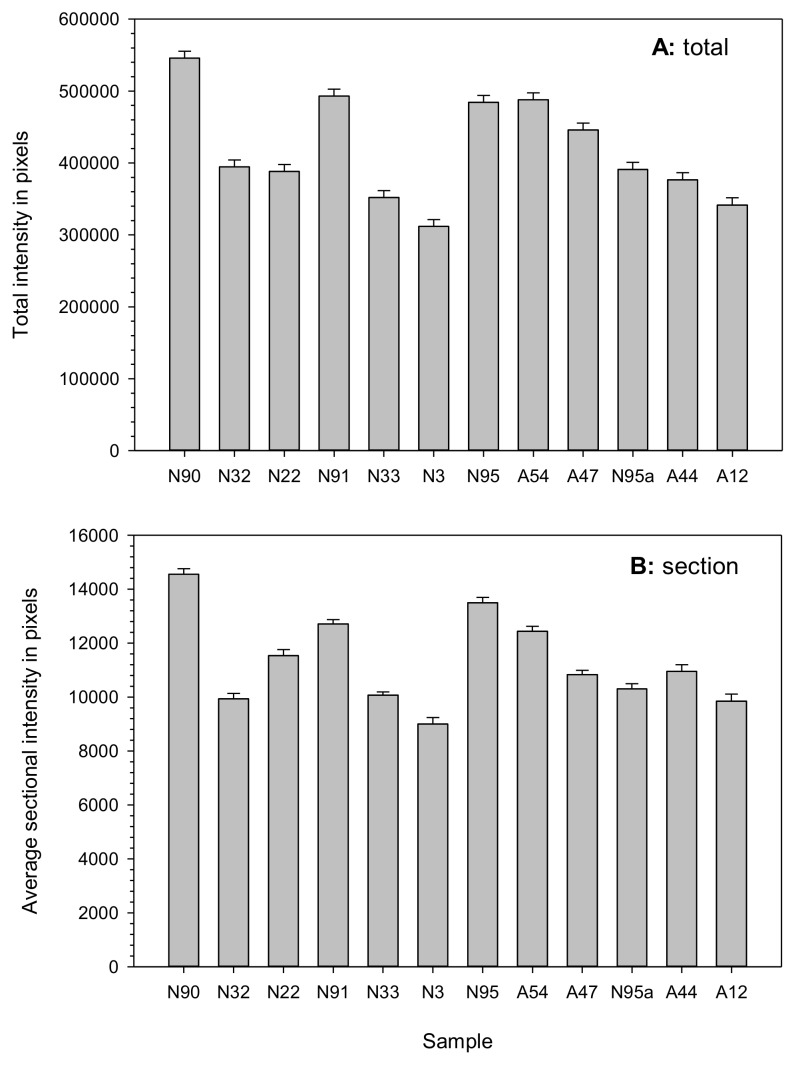
Total or sectional staining intensity measures showing higher intensities for the naturally and artificially aged seed samples with higher germination rates, with the one exception of sample N95a. The total (**A**) and average sectional (**B**) intensities were measured by ImageJ software. Each seed sample is labeled with its aging treatment (N for natural aging or A for artificial aging) and germination rate (see Table 1).

Assessing the fluorescence intensities of the various sections of the assayed seeds showed higher intensities in seed samples with higher germination rates, particularly in the NA seeds (Figure 4). For example, samples N90 and N91 had higher intensities than those of the degraded seeds of samples N22 and N3. However, an exception existed in seed sample N95a, which showed lower staining intensities than those of the AA seeds. Such patterns of staining intensity largely remain the same for total or sectional staining measures. Further linear regression analyses revealed statistically significant correlations between germination rate and total fluorescence intensity (*β* = 166529.9, *P* < 0.0031, *R*^2^ = 0.60) and between germination rate and sectional fluorescence intensity (*β* = 3642.5, *P* < 0.0070, *R*^2^ = 0.53).

### 2.3. DNA Assessment of Nuclear Alterations

**Figure 5:**
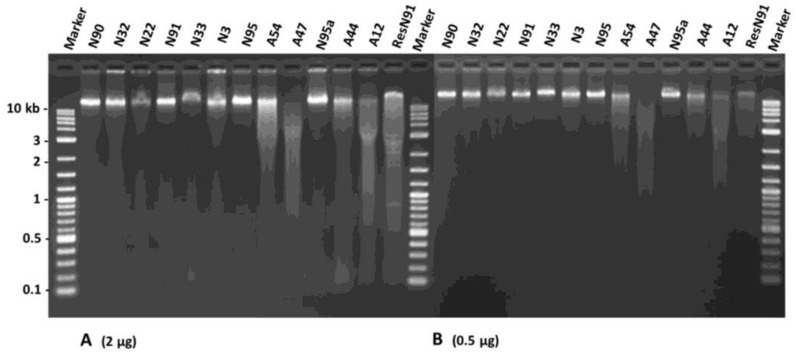
An agarose gel illustration of DNA alterations in DNA extracted from 12 aging flax seed samples with different germination levels. The brightness and mobility of the extracted DNA with equal amount ((**A**): 2 µg and (**B**): 0.5 µg) were shown for each sample. Each sample is labeled with its aging treatment and germination rate (see Table 1). ResN91 represents the DNA extracted from sample N91, which was digested by the *Eco*RI enzyme. Generally, DNA of the seed samples with lower germination rates displayed more smearing or more degradation, particularly after artificial aging.

To verify if DNA degradation had occurred in the aged flax seeds, a DNA assessment was conducted on 12 seed samples representing two aging treatments and germination levels (see Section 4.5 and Table 1). The DNA concentration per sample ranged from 142.5 to 302.7 ng/µL with an average of 210.1 ng/µL. The DNA purity per sample measured by 260/280 values ranged from 1.43 to 1.71 and averaged 1.56. These 260/280 values were smaller than 1.8, suggesting that the overall DNA purity of the assayed seed samples was lower than expected. However, such lower DNA purity did not seem to affect the quality of the assessment much. Loading 2 µg of total DNA per sample onto 1.5% agarose gel revealed a lower amount of high-molecular-weight DNA and more DNA smearing for samples with lower germination rates (Figure 5A). For example, sample N22 had a fainter band (or lower amount of high-molecular-weight DNA) than sample N90. Similarly, all four AA samples of low germination rate (A54, A47, A44, and A12) displayed marked DNA smearing. To confirm the quality assessment further, 500 ng of total DNA were loaded onto 1.5% agarose gel, and the intensities of the intact bands from all the seed samples were assessed (Figure 5B). More DNA smearing was observed in the AA seeds than in the NA seeds. More DNA smearing was also associated with seed samples with lower germination rates. These results indicate that DNA degradation had occurred in these aged seeds.

## 3. Discussion

Our experiment generated two sets of interesting results regarding aged flax seeds. First, the TUNEL assay and DAPI staining revealed few alterations of the cellular morphology of the naturally and artificially aged seeds of cultivated flax. Second, the revealed DNA degradations in the aged flax seeds appeared to be associated with the seed samples with low germination rates. These findings indicate that oily flax seed aging may alter cellular morphology differently than starchy wheat seed aging. The results also imply that TUNEL assay and DAPI staining may not yield informative assessments of cellular alterations and DNA degradation after the aging of oily seeds. However, the cause of the few alterations of the cellular morphology of the aged flax seeds remains unclear. It is possible that the cytoplasms of the cotyledon, radicle, and aleuronic cells of the aged flax seeds were filled with oil and protein bodies similar to those in rapeseeds [30], so that the TUNEL assay and DAPI staining had little resolution with which to detect the embryonic and cellular changes. 

The finding of few cellular alterations in the aged flax seeds was not expected, given that the same experiment was conducted on aged wheat seeds [28]. The aged wheat seeds displayed substantial changes in the scutellum but not the aleurone. Longer AA treatments on the wheat seeds resulted in the loss of the scutellum cell structure, the collapse of cell layers, and the disappearance of honeycomb arrangements. Our flax finding also differed from the findings of those experiments reported on the aged seeds of other plant species, such as sunflowers [25], elm [26], and peas [27]. For example, TUNEL assay coupled with DAPI staining was able to detect the nuclei of dead cells and embryonic structures in the paraffin-embedded sections of sunflower embryonic axes of unaged and aged sunflower seeds [25]. Thus, it is not clear how general the flax seed findings (Figure 1, Figure 2 and Figure 3) are with respect to oily seeds of other plant species. Future studies on other oily seeds are required. 

The findings reported here, along with those from the SSR analysis [29], also suggest caution in the search for specific seed aging signals as biomarkers for seed viability prediction. Clearly, selection of a seed aging signal for exploration has become a critical issue. A fruitful exploration of seed aging signals requires a better understanding of different potential aging signals, as not all aging signals are equally informative and sensitive [1]. Changes in RNA integrity [31] and ROS activity [26,27] might carry more sensitive aging signals than changes in cellular morphology. A thoroughly designed experiment with advanced tools may also need to detect the specific seed aging signals.

## 4. Materials and Methods

### 4.1. Plant Material

The flax seed samples used in this study are listed in Table 1, and they were obtained from the Plant Gene Resources of Canada (PGRC), Saskatoon Research and Development Center, Saskatoon, Canada. The flax material was comprised of seeds stored in the PGRC for various time periods under natural aging and displayed variable rates of germplasm. Artificial aging of the flax seeds was performed following the inner chamber ‘tray method’ and the published set-up procedure [32]. These seed samples received different time/temperature treatments to achieve varying germination rates (Table 1). Germination testing was performed following the Association of Official Seed Analysts guidelines [33]. The germination was assessed on three replicates of 100 seeds for each sample and control seed sample in the following three steps. First, germination papers were moistened with 45 mL distilled water, and seeds were placed between the first of three layers of those moistened papers. Second, the germination papers were rolled and placed in a plastic bag in a controlled environment incubator (Hoffman Manufacturing Inc. Albany, OR, USA) for 8 h of light and 16 h of darkness at 20 °C. Third, normally germinated seedlings were counted after seven days, and the percentage was calculated. Note that a seed sample was labeled with its aging treatment (N for naturally aged or A for artificial aging) in combination with its germination rate (%).

### 4.2. Seed Fixation, Processing, and Tissue Sectioning

Approximately 10 seeds from each seed sample were randomly selected without considering the germination of their seed sample and used for paraffin embedding. The selected seeds were treated overnight at room temperature in 2% glutaraldehyde and 2% formaldehyde in 50 mM phosphate buffer (PBS, pH 7.5) with 150 mM NaCl. Dehydration was performed with increasing concentrations of ethanol (50%, 70%, 80%, 90%, and 100%) on the treated seeds for 5 min and a final step with 100% ethanol for 10 min. The tissue was then left in 100% ethanol overnight at 4 °C. Following the overnight incubation, the tissue was incubated in 100% ethanol for 1 h at room temperature. After removing from the 100% ethanol, the tissue was incubated in 50% ethanol (50% Citrisolv (Fisher Scientific, Ottawa, ON, Canada)) solution for 4 h at room temperature and then incubated overnight in 100% Citrisolv at room temperature. Following the overnight incubation, fresh Citrisolv was added. Then, the tissue was submerged in a solution with 50:50 Citrisolv and Paraplast chips (SPI Supplies, West Chester, PA, USA) and left in a constant temperature oven (Yamato Scientific America, Inc., Santa Clara, CA, USA) at 60 °C. The Paraplast was changed daily over the following 2–4 days. Following the incubation, the tissue was embedded in paraffin wax (Parchem, New Rochelle, NY, USA). A mold box was placed on a warmer and filled with molten paraffin wax. The tissue was placed in the mold box in a desired orientation using pre-warmed forceps. To cool and solidify the paraffin wax, the mold box was placed in water overnight. The embedded issue was kept at 4 °C until further use. Before starting the sectioning of the embedded material, the additional wax was removed with the help of a razor blade to make small squares of wax containing single seeds only in the middle of the square. From each square, a section of 6 µm was cut by using a Leitz 1212 rotary microtome (Leica Biosystems, Concord, ON, Canada). Sections were cut in continuous ribbon form, and around 6–8 sections were generated per ribbon. Approximately two ribbons, or 16–18 sections, were glued to each pre-warmed poly-L-lysine-coated slide at 45 °C, each containing a few drops of water. The ribbon was carefully placed on water and left for 15 min, and then, excess water was removed by Kiwi paper. The slide was left on a warmer for at least 4 h and then kept at 4 °C until further use. 

### 4.3. TUNEL Assay, DAPI Staining, and Microscopy

The DeadEnd™ Fluorometric TUNEL System (Promega, Madison, WI, USA) was applied to detect DNA degradation in the cells following the manufacturer’s instructions. Briefly, slides were first soaked in Citrisolv solution for wax removal, followed by washes in a series of ethanol concentrations (100%, 95%, 85%, 70%, and 50%), each for 5 min. The slides were then washed in 0.85% NaCl and PBS, fixed in 8% formaldehyde and 8% glutaraldehyde in PBS, and treated with proteinase K^+^ (provided with the kit). Then, the TUNEL reaction mixture was added to the sections. The slides were incubated in a humidified chamber in the dark at 37 °C for 1 h, and the reaction was stopped in 2× SSC buffer with 3 washes in PBS. The dried slides were stained using Vectashield Mounting Medium with DAPI (4′,6-diamidino-2-phenylindole) (Vector Laboratories., Burlingame, CA, USA) staining solution and placed at 4 °C with a cover slip, protected from light before microscopy. The stained seed sections were observed under an Apotome fluorescence microscope (Zeiss, Germany). The following parameters were used to examine the fluorescent samples: excitation wavelength 365 nm, beam splitter FT-395, and emission filter 420–470 nm. The images were taken using a digital CCD camera (AxioCam MRm, black/white, 1.4 MP) mounted on the microscope. The resulting images were processed and analyzed using imaging software Zeiss AxioVision 4.8 (Zeiss, Germany). 

### 4.4. Analysis of Micrographs

First, flax seed micrographs were visually analyzed to assess the differences in (1) cell size, shape, and arrangement in a cell layer; (2) arrangements of cell layers in different sections; and (3) nucleus staining from different samples. Following the visual inspection, ImageJ software [34] was used to measure pixel differences in the nucleus staining. To assess the differences in the nucleus staining of flax cotyledons and radicles, 6–7 micrographs of each sample were analyzed, and the averaged intensities were represented. To evaluate the differences in the flax cotyledon or radicle intensities, different sections of a single slide were also observed using the same measuring square box for all samples. The generated data were represented as bar graph after arranging the data in ascending order for each seed.

### 4.5. Assessment of DNA Degradation

All 12 flax seed samples (Table 1) were chosen to assess the changes in the DNA quality in the NA or AA seeds. DNA was first extracted from the flax seeds using the Nucleo Spin^®^ Plant II kit (Macherey-Nagel, Düren, Germany) according to the manufacturer’s instructions, but the yield and quality were poor due to the high oil content. Additional efforts were then made to apply several DNA extraction methods. As a result, the application of the sodium dodecyl sulfate (SDS) method as described by Demeke et al. [35] generated workable DNA yield and quality. Six flax seeds were placed in a folded paper, crushed with pliers, and put in a 2 mL centrifuge tube with one tungsten carbide bead. The seeds were pulverized in a mixer mill for 1 min at 20 Hz before adding L mL of dry seed extraction buffer (200 mM Tris-HCl pH 7.5, 288 mM NaCl, 25 mM EDTA, 0.5% SDS). The samples were homogenized with the mixer mill for 30 s at 20 Hz and centrifuged for 5 min at 19,000× *g*. The supernatant was transferred to a new 2 mL centrifuge tube, and 215 µL of potassium acetate for alkaline lysis [36] was added and mixed with the supernatant before incubating on ice for 30 min. The samples were centrifuged at 14,000× *g* for 15 min at 4 °C, and then, the supernatant was transferred to a new 1.5 mL tube. Five microliters of RNaseA (10 mg/mL) were added to each tube before incubating for 15 min at 37 °C. After RNase treatment, 500 µL of isopropanol was added, and the samples were mixed by inversion before centrifuging for 2 min at 19,000× *g*. The pellets were washed with 1 mL of 70% ethanol and then dried in a Vacufuge Plus (Eppendorf Canada, Mississauga, ON, Canada) at 45 °C for 15 min. The DNA was re-suspended in 30 µL of 1× TE (10 mM Tris-HCl, 1.0 mM EDTA, pH 8.0). The DNA extraction was replicated three times for each seed sample.

The DNA purity was assessed by absorption using the 260/280-nm ratio from the Thermo Scientific Nanodrop 8000, and the DNA was quantified using an Invitrogen Quant-iTTM PicoGreen^®^ dsDNA Assay Kit (Life Technologies, Burlington, ON, Canada). The DNA quality was also evaluated by loading both 2 µg and 500 ng of genomic DNA from each sample onto a gel with 1.5% agarose. The gel was run for 2.5 h at 100 V in 1× TAE. A control was included using DNA from sample N91 digested with the *Eco*RI restriction enzyme for 3 h at 37 °C. These gels would help to illustrate the extent of intact DNA (or high-molecular-weight DNA) and DNA smearing for the determination of DNA degradation in the aged seeds.

## Figures and Tables

**Table 1:** Flax materials studied and information regarding their storage year, aging, germination, microscopy, sample label, and DNA assessment.

				Microscopy			Sample Label	DNA Assessment	
Sample	Year	Aging	GR	NS	NSO	PT		DNA(ng/µL)	260/280
CN101154	2000	Natural	90%	9	180	54	N90	302.7(74.6)	1.65(0.01)
CN98246	1998	Natural	32%	9	210	192	N32	187.6(34.6)	1.49(0.07)
CN98245	1998	Natural	22%	10	230	124	N22	144.1(28.5)	1.44(0.04)
CN96846	1998	Natural	91%	9	210	194	N91	193.8(48.4)	1.66(0.02)
CN98062	1998	Natural	33%	7	126	68	N33	142.5(6.6)	1.57(0.05)
CN33389	1973	Natural	3%	9	200	124	N3	205.5(26.9)	1.54(0.03)
CN116253	2014	Natural	95%	10	240	106	N95	287.4(62.7)	1.61(0.02)
CN116253	2014	Aged 96 h@43 °C	54%	9	190	55	A54	218.5(22.3)	1.53(0.05)
CN116253	2014	Aged 120 h@44 °C	47%	10	240	142	A47	221.0(20.3)	1.57(0.04)
CN116255	2014	Natural	95%	10	240	119	N95a	214.6(93.6)	1.71(0.26)
CN116255	2014	Aged 96 h@43 °C	44%	10	240	106	A44	198.5(22.5)	1.43(0.01)
CN116255	2014	Aged 120 h@44 °C	12%	10	240	100	A12	204.9(35.5)	1.55(0.03)
Total				112	2546	1384			

Note: CN represents the Canadian accession number used in the Plant Gene Resources of Canada (PGRC) flax collection; Year, the year for the materials initially stored; Natural, natural aging in the PGRC collection; Aged, artificial aging (AA) under different time and temperature; GR, germination rate obtained between 2014 and 2015; NS, number of seeds embedded in paraffin for TUNEL and DAPI staining; NSO, number of sections observed under microscope; and PT, number of pictures taken. Each sample is labeled with aging method (N for natural aging and A for artificial aging) plus its germination rate. N95 and N95a are control samples before AA. DNA concentration and purity per sample are shown as an average and its standard deviation is in parenthesis.
